# Endothelial Phenotype Evoked by Low Dose Carvedilol in Pulmonary Hypertension

**DOI:** 10.3389/fcvm.2018.00180

**Published:** 2018-12-12

**Authors:** Hoi I Cheong, Samar Farha, Margaret M. Park, James D. Thomas, Didem Saygin, Suzy A. A. Comhair, Jacqueline Sharp, Kristin B. Highland, W. H. Wilson Tang, Serpil C. Erzurum

**Affiliations:** ^1^Lerner Research Institute, Cleveland Clinic, Cleveland, OH, United States; ^2^Heart and Vascular Institute, Cleveland Clinic, Cleveland, OH, United States; ^3^Heart and Vascular Institute, Northwestern University Hospital, Chicago, IL, United States; ^4^Respiratory Institute, Cleveland Clinic, Cleveland, OH, United States

**Keywords:** pulmonary hypertension, carvedilol, β-blockers, nitric oxide, right ventricle

## Abstract

**Background:** The therapeutic benefits of β-blockers are well established in left heart failure. The Pulmonary Arterial Hypertension Treatment with Carvedilol for Heart Failure [PAHTCH] study showed safety and possible benefit of carvedilol in pulmonary arterial hypertension (PAH) associated right heart failure over 6 months. This study aims at evaluating the short-term cardiovascular effects and early mechanistic biomarkers of carvedilol therapy.

**Methods:** Thirty patients with pulmonary hypertension (PH) received low dose carvedilol (3.125 mg twice daily) for 1 week prior to randomization to placebo, low-dose, or dose-escalating carvedilol therapy. Echocardiography was performed at baseline and 1 week. Exercise capacity was assessed by 6 min walk distance (6MWD). The L-arginine/nitric oxide pathway and other biological markers of endothelial function were measured.

**Results:** All participants tolerated 1 week of carvedilol without adverse effects. After 1 week of carvedilol, 6MWD and heart rate at peak exercise did not vary (both *p* > 0.1). Heart rate at rest and 1 min post walk dropped significantly (both *p* < 0.05) with a trend for increase in heart rate recovery (*p* = 0.08). Right ventricular systolic pressure (RVSP) decreased by an average of 13 mmHg (*p* = 0.002). Patients who had a decrease in RVSP of more than 10 mm Hg were defined as responders (*n* = 17), and those with a lesser drop as non-responders (*n* = 13). Responders had a significant drop in pulmonary vascular resistance (PVR) after 1 week of carvedilol (*p* = 0.004). In addition, responders had a greater decrease in heart rate at rest and 1 min post walk compared to non-responders (both *p* < 0.05). Responders had higher plasma arginine and global bioavailability of arginine at baseline compared to non-responders (*p* = 0.03 and *p* = 0.05, respectively). After 1 week of carvedilol, responders had greater increase in urinary nitrate (*p* = 0.04). Responders treated with carvedilol had a sustained drop in RVSP and PVR after 6 months of carvedilol with no change in cardiac output.

**Conclusions:** Low-dose carvedilol for 1 week can potentially identify a PH responder phenotype that may benefit from β-blockers that is associated with less endothelial dysfunction.

**Clinical Trial Registration:** http://www.clinicaltrials.gov. identifier: NCT01586156.

## Introduction

Pulmonary arterial hypertension (PAH) is a progressive pulmonary vascular disease defined by elevated pulmonary arterial pressure leading to right-sided heart failure. Recent studies have investigated the safety and potential benefits of β-blockers in pulmonary arterial hypertension (PAH) related heart failure (1, 2). Whereas, the therapeutic benefits of β-blockers are well established in left-sided heart failure (3, 4); studies in PAH have shown conflicting results. In a small study, 10 patients with portopulmonary hypertension on β-blockers for reasons other than PAH, were found to have an increase in 6 min walk distance and cardiac output (CO) after β-blocker withdrawal (5). Another case report described a single patient with portopulmonary hypertension treated with β-blockers for supraventricular tachycardia leading to acute cardiovascular decompensation (6). On the other hand, other studies support the use of β-blockers in PAH. In a retrospective analysis, PAH patients on β-blockers for conditions unrelated to PAH had similar survival and functional outcomes compared to those not receiving β-blockers (7). In a prospective non-randomized cohort study of 94 patients, β-blockers were reported to be safe in PAH, as defined by pulmonary hemodynamic, functional, or cardiac outcomes over 2 months (8). In another small pilot study, carvedilol was found to be safe in patients with stable PAH and right ventricular (RV) dysfunction and was associated with an improvement in RV ejection fraction (9). A randomized prospective small clinical trial showed bisoprolol therapy to be well tolerated in PAH patients but there was an associated decrease in cardiac index and 6 min walk distance (10). Recently, a double-blind, randomized, controlled trial of Pulmonary Arterial Hypertension Treatment with Carvedilol for Heart Failure (PAHTCH) showed that carvedilol is safe over 6 months of therapy and has clinical and mechanistic benefits associated with improved outcomes (11). The PAHTCH trial design included a 1 week run-in, followed by randomization of participants to one of three arms for 24 weeks: placebo, low-fixed dose, or dose-escalating carvedilol (11).

Prior studies of the short-term effects of β-blockade did not evaluate cardiac function and/or pulmonary pressures, or biologic markers of disease pathway activation. The L-arginine/nitric oxide (NO) pathway is abnormal in PAH. L-arginine, the substrate for synthesis of the vasodilator NO, is consumed by arginase to produce ornithine and urea, and/or altered by methylation pathways that inhibit NO production (12). In PAH, plasma arginase activity levels are higher than normal, and the global bioavailability of arginine for NO synthesis, determined by the ratio of arginine to ornithine and citrulline, is lower in PH patients (13–15). High arginase activity and concomitant low NO synthesis are associated with greater disease severity and higher mortality (13–15). Likewise, arginine methylation is dysregulated with increased asymmetric dimethylarginine (ADMA), an inhibitor of NO synthetase (15). Cyclic guanosine monophosphate (cGMP) is the downstream effector of NO, but also is part of the signaling pathway for brain natriuretic peptide (BNP), a hormone released by the straining heart that mediates beneficial natriuresis and vasodilation (16). The pro-hormone, pro-BNP, is cleaved to BNP and an inactive metabolite N-terminal pro-BNP (NT-proBNP). Currently, BNP or NT-proBNP are the only biomarkers recommended by clinical guidelines to monitor disease and address treatment goals (17). While the L-arginine/NO and BNP pathways activate guanylate cyclase, resulting in the release of cGMP that mediates vasodilation (17), endothelial release of endothelin-1, a vasoconstrictor, is excessive in PAH, and blocking the endothelin receptor is a successful strategy for therapy (18).

Here, the cardiovascular effects of short-term (1 week) use of carvedilol at low dose were evaluated in patients with PH, in order to assess clinical and biological markers that might identify those with an acute beneficial response to β-blockers. To study the short-term effects of carvedilol, the PAHTCH cohort was analyzed for clinical characteristics and echocardiographic measures as well as biomarkers of endothelial function, including the arginine/NO pathway, BNP, and endothelin-1 at baseline prior to drug and at 1 week of low-dose carvedilol. In contrast to the prior PAHTCH report, this study provides new information on the 1 week low-dose carvedilol intervention evaluating both clinical measures and mechanistic biomarkers.

## Methods

### Study Design and Participants

The Pulmonary Arterial Hypertension Treatment with Carvedilol for Heart Failure (PAHTCH) clinical trial was approved by the Institutional Review Board at the Cleveland Clinic. A total of 30 PH patients and 12 healthy participants were consented and enrolled. PH patients started with a 1 week open-label low-dose carvedilol at 3.125 mg twice daily, followed by double-blinded randomization to placebo, low-fixed dose or escalating dose arms. Study design and participants have been previously described in detail (11).

### Echocardiogram

All echocardiograms and Doppler examinations were performed on a General Electric (GE) Ultrasound system (models E95 and E9), by an advanced cardiac sonographer (MP). Analysis was performed offline via Syngo Dynamics Cardio PACS system, Siemens Healthcare and EchoPAC Clinical workstation, GE Medical Imaging. Transthoracic echocardiograms were performed after 1 week of carvedilol to assess acute changes in cardiac structure and function as safety measures (11). For the purpose of this study, echocardiograms were later retrieved to collect parameters not quantified in real time. Three data points were analyzed and the average reported with the exception of the peak TR velocity. High frame imaging between 60 and 90 frames per second was acquired for both left ventricular (LV) and right ventricular (RV) global peak systolic longitudinal strain analysis. Longitudinal strain for the left and right ventricle was measured off line using GE EchoPAC software. The aortic valve opening and closure time was set using the aortic valve pulsed wave Doppler trace acquired from the left ventricular outflow tract. From an apical 4-chamber view focusing on the LV, data points marking the septal, and lateral mitral valve annulus and the apex were joined to trace the entire LV myocardial border. The speckle tracking thickness was adjusted to match the myocardial thickness. The image was then played continuously to check that tracking of all wall segments was appropriate. When necessary, adjustments in tracking were made. If adjustments were necessary more than twice, a new image clip was selected for analysis.

The same method was applied to the RV for analysis of peak global RV longitudinal strain. The spectral Doppler waveform recorded at the level of the right ventricular outflow tract was used to identify the timing of pulmonic valve opening and closing times. An RV focused, or modified view was captured during breath hold and analyzed. RV peak global RV longitudinal strain values for the base, mid and apical RV lateral wall segments were recorded as well as the global 6-wall strain, which also included the interventricular base, mid, and apical segments.

Continuous wave (CW) Doppler of the peak tricuspid valve regurgitant (TR) jet was used to calculate RV systolic pressure using the Bernoulli equation. For weak and insufficient TR jets, agitated saline contrast was used to enhance the TR jet signal. A peak TR Doppler jet of good quality was obtainable on all study subjects. Right atrial pressure was calculated from the change in inferior vena cava collapse during respiration and with the sniff test as reported. Two-dimensional echo analysis included LV end diastolic and systolic diameters measured from the parasternal long axis view, LV end diastolic and systolic volume assessment (biplane method of disc) for LV ejection fraction (LVEF), cardiac output (CO), and stroke volume (SV). The pulmonary vascular resistance (PVR) was calculated by: Peak TV velocity/time velocity integral of the RVOT x 10 + 0.16 = PVR in Woods (19).

### NT-proBNP

Plasma NT-proBNP was measured by an electrochemiluminescence immunoassay using the Roche Cobas e411 analyzer as previously described (11). Data <5 pg/mL are below detectable level and are assigned an arbitrary value of 4. NT-proBNP was measured at baseline and 3 and 6-months visits.

### Arginine Metabolism

Measurements of arginine, ornithine, citrulline, asymmetric dimethylarginine (ADMA), symmetric dimethyl arginine (SDMA), and monomethyl arginine (MMA) were performed by stable-isotope-dilution High-performance liquid chromatography (HPLC) with online tandem mass spectometry as previously described (20). These markers were measured at baseline and 3 and 6-months visits.

### Urinary Nitrate and cGMP

All values of urine samples were normalized by urine creatinine to account for differences in hydration and urine volume. Creatinine concentrations were measured with the Abbott Architect machine according to manufacturer's instructions. All samples tested negative for nitrite using the Griess reaction. Nitrate (NO${}_{3}^{-}$) was measured by chemiluminescence as previously described (21). cGMP was quantified by diluting urine samples by 10-fold according to the manufacturer's protocol of the cGMP parameter assay (KGE003, R&D Systems, Minneapolis, MN). Urinary nitrates and cGMP were measured at all visits.

### Serum Endothelin-1

Serum endothelin-1 was measured by the Endothelin-1 Quantikine ELISA Kit (DET100, R&D Systems, Minneapolis, MN) according to the manufacturer's protocol at baseline and 3 and 6-months visits.

### Statistical Analysis

All analyses were performed using JMP Pro, version 13.1 (SAS Institute). One-tail Fisher's Exact (for 2 × 2 tables) or Likelihood Ratio chi-square test was used for comparison of categorical variables. One-tail Student's *t*-test was used for quantitative values with natural log transformation used for values with skewed distributions. Associations among quantitative measures were assessed using Pearson correlations. Statistical significance was accepted at a level of *p* ≤ 0.05.

## Results

### Baseline Characteristics

**Figure 1:**
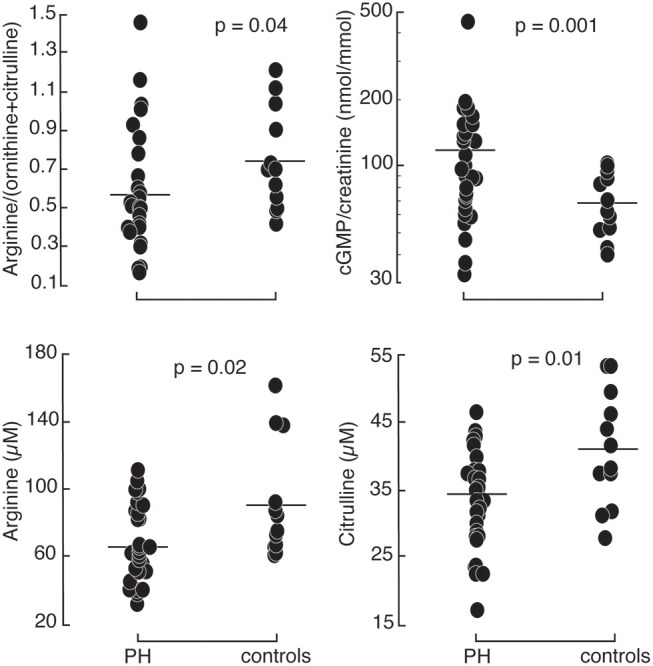
Comparison between healthy controls (*N* = 12) and PH subjects (*N* = 30): PH patients had higher urinary cyclic guanosine monophosphate normalized by creatinine (cGMP/creatinine). They had lower arginine, citrulline, and global arginine bioavailability compared to healthy controls.

Patients with pulmonary hypertension (*N* = 30) and healthy controls (*N* = 12) were enrolled in the study. The study population was described in a prior report (11). The majority of the patients were WHO group 1 PAH (*N* = 26) with 2 patients with chronic thromboembolic PH (WHO group 4) and 2 with PH associated with lung diseases (WHO group 3). Among the 30 PH participants, 24 were taking phosphodiesterase type 5 inhibitors, 19 endothelin receptor antagonists, and 16 prostacyclin analogs. Healthy controls and PH patients were ethnically similar. There were no differences in age or gender distribution. However, PH patients had overall higher body mass index and lower oxygen saturation. Indicators of RV and pulmonary vascular function as measured by right ventricular systolic pressure (RVSP), plasma NT-proBNP and endothelin-1 were higher in PH patients compared to controls. In addition, urinary cGMP was higher in PH compared to healthy controls. Plasma arginine levels and global arginine bioavailability (ratio of arginine to ornithine and citrulline) and citrulline were lower in PH, whereas the plasma level of MMA, an endogenous inhibitor of nitric oxide synthase, was higher (Table 1; Figure 1). No differences in endothelial markers were observed as measured by plasma levels of ornithine, SDMA, and ADMA, and in urinary nitrate between healthy controls and PH patients in this study (Table 1). These findings support the abnormalities in endothelial NO pathway in PH.

**Table 1:** Baseline biomarkers of endothelial function.

**Characteristics**	**Healthy controls (*n =* 12)**	**PH (*n* = 30)**	***t*-test *p*-value**
Arginine (μM)	91 ± 35	66 ± 23	0.02
Ornithine (μM)	76 ± 22	85 ± 28	0.2
Citrulline (μM)	41 ± 9	34 ± 7	0.01
Arginine/(ornithine + citrulline)	0.74 ± 0.26	0.57 ± 0.30	0.04
MMA (μM)	0.11 ± 0.04	0.14 ± 0.04	0.02
SDMA (μM)	0.64 ± 0.11	0.62 ± 0.20	0.3
ADMA (μM)	0.52 ± 0.07	0.55 ± 0.14	0.2
Urine nitrate/creatinine (μmol/μmol)	0.08 ± 0.04	0.08 ± 0.06	0.5
Urine cGMP/creatinine (nmol/mmol)	68 ± 19	120 ± 79	0.001
Endothelin-1 (pg/mL)	0.92 ± 0.25	3.07 ± 1.52	< 0.0001

*PH, pulmonary hypertension; MMA, monomethyl arginine; SDMA, symmetric dimethyl arginine; ADMA, asymmetric dimethylarginine; cGMP, cyclic guanosine monophosphate*.

*Data shown as mean ± SD, one-sided t-test unequal variance*.

Since cGMP is the downstream signaling molecule of both NO and BNP pathways, we tested the strength of relationship of these biomarkers in PH patients and healthy controls. At baseline, plasma NT-proBNP directly correlated with urine cGMP/creatinine in healthy controls (*R*^2^ = 0.5; *p* = 0.007) and in PH (*R*^2^ = 0.4; *p* = 0.0004) (Supplemental Figure 1). Likewise, urinary nitrate correlated with urinary cGMP in healthy controls (*R*^2^ = 0.8; *p* = 0.0002) and in PH (*R*^2^ = 0.5; *p* < 0.0001; Supplemental Figure 1). In addition, cGMP related to clinical markers of disease severity. In fact, urinary cGMPcreatinine positively correlated with RVSP in healthy controls (*R*^2^ = 0.8; *p* = 0.0003) and in PH (*R*^2^ = 0.2; *p* = 0.02). Similarly, plasma NT-proBNP directly correlated with RVSP in PH (*R*^2^ = 0.2; *p* = 0.009l; Supplemental Figure 2). Urinary nitrate/creatinine and cGMP/creatinine both inversely correlated with 6 min walk distance in PH (Nitrate: *R*^2^ = 0.2; *p* = 0.03; cGMP: *R*^2^ = 0.3; *p* = 0.002; Supplemental Figure 2).

### Changes in Clinical Parameters in PH Patients After 1 Week of Carvedilol

All patients tolerated 1 week of carvedilol at 3.125 mg twice daily. Exercise capacity as measured by 6 min walk distance did not vary after 1 week of low dose carvedilol. Heart rate at rest dropped by an average of 6 beats/minute (paired *t-*test *p* = 0.01). Heart rate at maximum exertion at 6 min did not vary on carvedilol therapy (*p* = 0.3); however heart rate at 1 min recovery post walk dropped significantly [Heart rate (beats/minute): baseline 98 ± 15; 1 week 87 ± 16; paired *t-*test *p* = 0.004] and heart rate recovery (HRR) tended to increase [HRR (beats/minute): baseline 20 ± 18; 1 week 27 ± 15; paired *t-*test *p* = 0.08].

**Figure 2:**
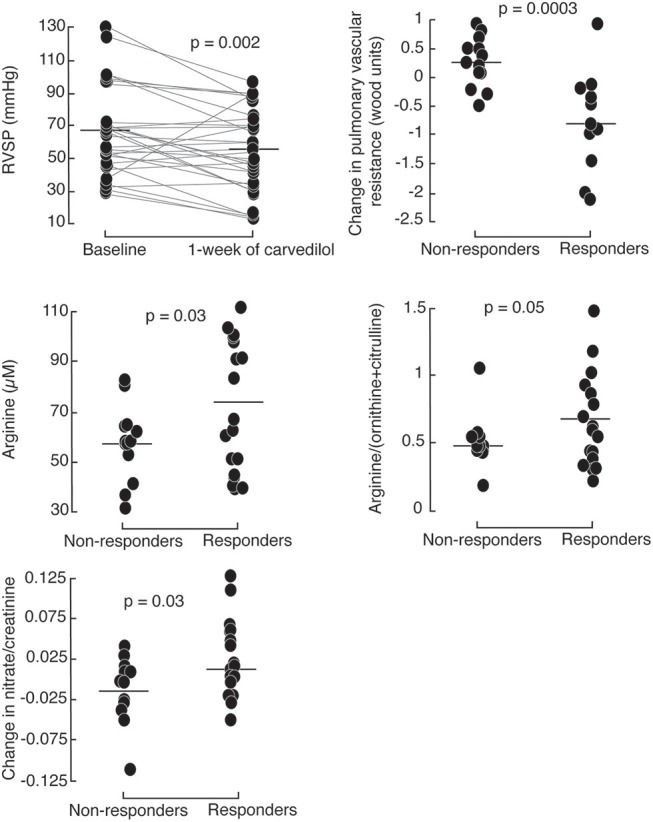
Right ventricular systolic pressure (RVSP) drops in PH patients after 1 week of 3.125 mg of carvedilol treatment. Responders, defined by a decrease of >10 mmHg RVSP after 1-week carvedilol treatment have higher baseline plasma arginine levels and global arginine bioavailability compared to non-responders. Responders have a greater increase in urinary nitrates compared to non-responders.

To further evaluate the short-term effect of low-dose carvedilol on cardiac function in PH, we analyzed different echocardiographic parameters of right and left ventricular function. After 1 week of low dose carvedilol 3.125 mg twice daily, RVSP dropped significantly in PH patients [RVSP (mmHg): baseline 67 ± 26; 1 week 54 ± 25; paired *t-*test *p* = 0.002; Figure 2). More than half of PH patients (*n* = 17) had a drop in RVSP of ≥ 10 mmHg, and were defined as responders (Supplemental Figure 3). Those with a lesser drop (RVSP < 10 mmHg) were defined as non-responders (*n* = 13). Responders had significantly lower RVSP at 1 week compared to non-responders [RVSP (mmHg): responders (*n* = 17) 46 ± 28; non-responders (*n* = 13) 65 ± 18; *p* = 0.01] and the change in RVSP from baseline was significant [change in RVSP from baseline (mmHg): responders −26 ± 10, non-responders 5 ± 16; *p* < 0.0001; Table 2; Figure 2]. Furthermore, responders had a more significant drop in heart rate at rest [Heart rate at rest (beats/minute): baseline 81 ± 8; 1 week 74 ± 6; paired *t-*test *p* = 0.02; Table 2].

**Table 2:** Characteristics of responders and non-responders at baseline and 1-week carvedilol.

	**Baseline**			**1-week carvedilol**		
	**Responders**	**Non-responders**	***t*-test *p*-value**	**Responders**	**Non-responders**	***t*-test *p*-value**
**Variable**	***N* = 17**	***N* = 13**		***N* = 17**	***N* = 13**	
Weight (kg)	76 ± 18	91 ± 19	0.02	76 ± 18	91 ± 19	0.02
Systolic blood pressure (mmHg)	116 ± 19	117 ± 13	0.4	111 ± 14	113 ± 14	0.4
Diastolic blood pressure (mmHg)	71 ± 12	72 ± 12	0.4	66 ± 12*	72 ± 10	0.08
Oxygen saturation (%)	96.8 ± 2.8	95.7 ± 2.0	0.1	96.8 ± 2.6	95.2 ± 2.6	0.06
**6-MINUTE WALK TEST**						
Distance walked (feet)	1547 ± 421	1468 ± 337	0.3	1510 ± 446	1476 ± 338	0.4
Oxygen saturation at 6 min (%)	89 ± 7	91 ± 4	0.1	88 ± 5	87 ± 8	0.4
Heart rate at rest (beats/min)	81 ± 8	79 ± 7	0.3	74 ± 6*	74 ± 13	0.5
Heart rate at 6 min (beats/min)	115 ± 19	123 ± 20	0.1	110 ± 21	120 ± 15	0.06
Heart rate 1 min post exercise (beats/min)	95 ± 15	101 ± 14	0.1	83 ± 16*	92 ± 14*	0.06
Heart rate recovery (beats/min)	19 ± 18	22 ± 19	0.4	26 ± 16	28 ± 15	0.4
**ECHOCARDIOGRAM**						
LVEDD (cm)	4.4 ± 0.6	4.6 ± 0.7	0.3	4.6 ± 0.9	4.6 ± 0.8	0.4
LVESD (cm)	2.6 ± 0.9	2.6 ± 0.5	0.5	2.7 ± 0.6	2.9 ± 0.8	0.3
LVEDV (ml)	92 ± 19	100 ± 33	0.2	84 ± 22	84 ± 26*	0.5
LVESV (ml)	40 ± 13	44 ± 16	0.2	37 ± 9	38 ± 18	0.4
LVEF (%)	61 ± 9	56 ± 6	0.05	55 ± 7*	56 ± 9	0.4
RAP (mmHg)	6 ± 3	8 ± 5	0.2	6 ± 3	8 ± 5	0.1
RVSP (mmHg)	71 ± 30	61 ± 20	0.1	48 ± 28*	65 ± 18	0.01
LV stroke volume (ml)	64 ± 18	61 ± 18	0.3	47 ± 16*	46 ± 10*	0.4
LV cardiac output (l/min)	4.8 ± 1.6	4.7 ± 1.5	0.4	3.1 ± 1.2*	3.1 ± 0.7*	0.5
Pulmonary vascular resistance (wood units)	3.1 ± 1.5	2.5 ± 0.7	0.08	2.3 ± 0.8*	2.7 ± 0.7*	0.08
LV peak global longitudinal strain (%)	−18 ± 3	−18 ± 3	0.5	−18 ± 4	−18 ± 2	0.3
RV peak global longitudinal strain (%)	−16 ± 4	−15 ± 5	0.3	−16 ± 3	−16 ± 4	0.4
RV fractional area change (%)	31 ± 9	27 ± 11	0.2	33 ± 10	33 ± 6*	0.5

*Data shown as mean ± SD; one-sided t-test unequal variance*.

* *p ≤ 0.05 for change compared to baseline*.

Overall, the mean cardiac output decreased with carvedilol [CO (L/minute): baseline 4.8 ± 1.6, 1 week 3.1 ± 1.0; paired *t-*test *p* < 0.0001]. Heart rate measured during echocardiography was not significantly reduced (*p* = 0.4) but stroke volume was decreased compared to baseline (*p* < 0.0001). Systolic and diastolic blood pressures dropped with carvedilol therapy [systolic blood pressure (mmHg): baseline 116.8 ± 16.8, 1 week 112.2 ± 13.8; paired *t-*test *p* = 0.03 and diastolic blood pressure (mmHg): baseline 71.4 ± 11.9, 1 week 68.7 ± 11.2; paired *t-*test *p* = 0.05]. Pulmonary vascular resistance (PVR) did not change significantly (paired *t-*test > 0.1). The drop in stroke volume was associated with a decrease in left ventricle end diastolic volume [LVEDV (ml): baseline 96 ± 26, 1 week 84 ± 23; paired *t-*test *p* = 0.01]. There was a non-significant drop in LV ejection fraction (LVEF) [LVEF (%): baseline 59 ± 8, 1 week 55 ± 8; paired *t-*test *p* = 0.05]. Despite these changes, LV and RV strains did not vary significantly (both *p* > 0.1). Both responders and non-responders had a decrease in CO [CO (L/min): responders baseline 4.8 ± 1.6, 1 week 3.1 ± 1.2; paired *t-*test *p* = 0.0001 and non-responders baseline 4.7 ± 1.5, 1 week 3.1 ± 0.7; paired *t-*test *p* = 0.003] [Table 2]. Systolic blood pressure tended to drop after 1 week of carvedilol non-responders (*p* = 0.08; Table 2). Diastolic blood pressure dropped significantly in responders (*p* = 0.03) (Table 2). There was no significant drop in heart rate as measured at time of echocardiography in either subgroup (both *p* > 0.05). In non-responders, the drop in CO was associated with a drop in left ventricular end diastolic volume [LVEDV (ml): baseline 100 ± 33, 1 week 84 ± 26; paired *t-*test *p* = 0.008] and a non-significant increase in PVR [PVR (Woods unit): baseline 2.5 ± 0.7, 1 week 2.7 ± 0.7; paired *t-*test *p* = 0.05]. The drop in LVESV was not significant (*p* = 0.09). There was no change in LVEF. On the other hand, in the responder group, the drop in CO was associated with a drop in PVR [PVR (Woods unit): baseline 3.1 ± 1.5, 1 week 2.3 ± 0.8; paired *t-*test *p* = 0.004] and a decrease in LVEF [LVEF (%): baseline 61 ± 9, 1 week 55 ± 7; paired *t-*test *p* = 0.008). Again, there were no changes in RV and LV strain with carvedilol in either group (all *p* > 0.1). The findings suggest a negative inotropic effect with short-term carvedilol with differential effect on the ventricles unique to responders and non-responders.

We further compared responders and non-responders at baseline. Both groups had similar age [Age (years): responders 42 ± 13, non-responders 46 ± 11; *t-*test *p* = 0.2]. There were no differences in gender, race, or WHO classification between responders and non-responders. Functional class as measured by New York Heart Association (NYHA) classification was not different between the 2 groups [NYHA class (I/II/III): responders 4/9/3, non-responders 0/10/3; *p* = 0.07]. There was no difference in PH therapy at baseline between the 2 groups (*p* > 0.1). Baseline biomarkers of disease severity, NT-proBNP and endothelin-1 were similar [NT-proBNP (pg/ml): responders 569 ± 1645, non-responders 464 ± 751; *p* = 0.3 and endothelin-1 (pg/ml): responders 2.6 ± 0.9, non-responders 3.6 ± 2.0; *p* = 0.1]. There were no significant differences between the 2 groups at baseline based on 6 min walk distance or echocardiography (Table 2).

### L-Arginine-Nitric Oxide Pathway in PH Responders

To identify the possible mechanisms of action of carvedilol, we compared features between responders and non-responders [Table 3]. At baseline, responders had higher plasma levels and global bioavailability of arginine compared to non-responders (Figure 2). Both responders and non-responders had similar levels of methylarginines, ADMA and MMA. However, responders tended to have lower SDMA levels compared to non-responders [SDMA (μM): responders 0.58 ± 0.17, non-responders 0.68 ± 0.21; *p* = 0.09]. After 1 week of low-dose carvedilol treatment, urinary nitrate/creatinine increased in responders compared to non-responders [Change in urinary nitrate/creatinine (μmol/μmol): responders 0.014 ± 0.046, non-responders−0.015 ± 0.040; *p* = 0.03]. These findings suggest that responders had recovery of L-arginine–nitric oxide pathway whereas non-responders did not.

**Table 3:** L-arginine-nitric oxide pathway of responders and non-responders at baseline.

**Characteristics**	**Responders (*n* = 17)**	**Non-responders (*n* = 13)**	***t*-test *p*-value**
Arginine (μM)	73 ± 26	58 ± 15	0.03
Ornithine (μM)	84 ± 30	86 ± 26	0.4
Citrulline (μM)	35 ± 8	34 ± 6	0.3
Arginine/(ornithine + citrulline)	0.64 ± 0.35	0.47 ± 0.21	0.05
MMA (μM)	0.13 ± 0.04	0.14 ± 0.04	0.2
SDMA (μM)	0.57 ± 0.17	0.68 ± 0.21	0.07
ADMA (μM)	0.52 ± 0.11	0.59 ± 0.16	0.1
Urine nitrate/creatinine (μmol/μmol)	0.079 ± 0.071	0.078 ± 0.033	0.5
Urine cGMP/creatinine (nmol/mmol)	131 ± 96	105 ± 51	0.2

*MMA, monomethyl arginine; SDMA, symmetric dimethyl arginine; ADMA, asymmetric dimethylarginine; cGMP, cyclic guanosine monophosphate*.

*Responders refer to PH patients whose right ventricular systolic pressure dropped >10 mmHg after 1-week of 3.125 mg carvedilol twice daily*.

*Data shown as mean ± SD, one-sided t-test*.

### Long-Term Follow up at 3 and 6-Months

**Figure 3:**
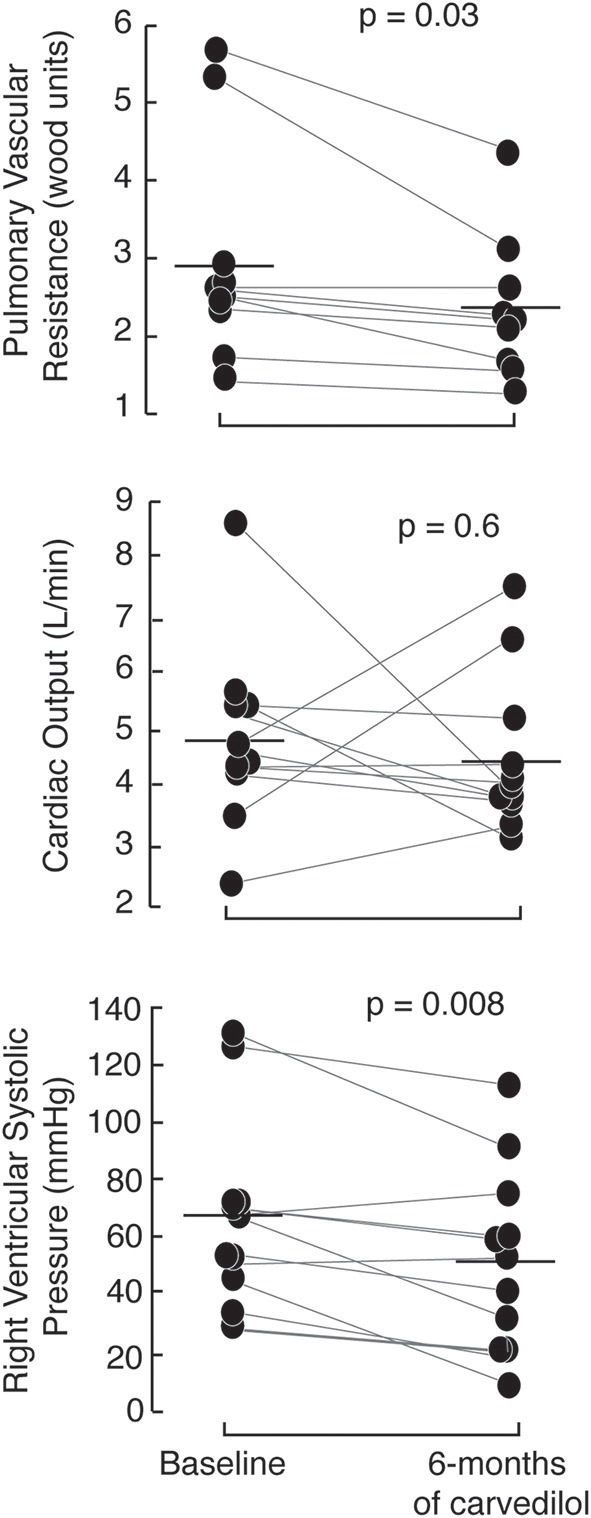
Short-term effects of carvedilol are sustained at 6-months of therapy with carvedilol. Responders, randomized to receive carvedilol either low-fixed or escalating dose of carvedilol have a marked drop in right ventricular systolic pressure and pulmonary vascular resistance (measured by echocardiography) at 6-months of therapy with no change in cardiac output.

The PAHTCH study design offered an ideal opportunity to determine if the early response to carvedilol predicted sustained response over time. After 1 week open-label carvedilol, 5 of the 17 PH responders were randomized to the placebo arm, 7 to the low-fixed dose carvedilol and 5 to the escalating dose carvedilol. Of the 13 non-responders, 5 were randomized to the placebo arm, 3 to the low-fixed dose carvedilol and 5 to the escalating dose carvedilol. There were no significant differences in the clinical parameters between responders and non-responders assigned to carvedilol at the 3 and 6-months visits. Responders on carvedilol had a sustained response to carvedilol at 6 months. In fact, RVSP and PVR were lower in responders at 6 months compared to baseline with no change in cardiac output (Figure 3). In addition, responders on carvedilol tended to increase RV strain at 6 months [RV strain (%): baseline −17 ± 2, 1 week −18 ± 3; paired *t-*test *p* = 0.05]. Responders on carvedilol at 6 months had higher arginine and arginine biovailability compared to non-responders on carvedilol [arginine (uM): responders 78.4 ± 21.7, non-responders 14.8 ± 5.2; *p* = 0.01 and arginine/(ornithine+citrulline): responders 0.63 ± 0.27, non-responders 0.45 ± 0.20; *p* = 0.05]. Responders on carvedilol had lower SDMA and ADMA at 6 months of therapy compared to non-responders (*p* = 0.01 and *p* = 0.006, respectively). These results suggest that the drop in RVSP after 1 week of therapy might predict patients who will maintain a drop in RVSP over time and that the response is related in part to recovery of the endothelial arginine/NO pathway.

## Discussion

Short-term carvedilol at low dose decreased pulmonary pressures in patients with pulmonary hypertension. Responders, who had a drop of more than 10 mmHg in RVSP after 1 week of therapy and continued carvedilol for the following 6 months, maintained a lower RVSP and PVR compared to non-responders on carvedilol. Further, responders had a greater decrease in heart rate at rest. Short-term carvedilol could potentially identify a responder subgroup that will maintain the decrease in RVSP overtime based on initial drop in RVSP and heart rate response. Independent of the possible therapeutic opportunity, the differential response to carvedilol also identifies distinct phenotypes of pulmonary hypertension.

The short-term effect of carvedilol was associated with increase L-arginine mediated NO production. In fact, responders to carvedilol had higher arginine levels and greater bioavailability of arginine at the beginning of the study as compared to non-responders. Carvedilol increased total body NO production, as measured by urinary nitrate, in responders but not in non-responders, indicating a potential mechanism of carvedilol *via* the L-arginine-nitric oxide pathway. This finding is consistent with a previous study of primary cultures of pulmonary arterial endothelial cells from explanted lungs of patients with PAH (22, 23). In that study, β-blockers recovered NO synthase (NOS) activity through reversal of phosphorylation-inactivation of the endothelial NOS. Deficiency of NO synthesis is a hallmark of PAH and implicated in its pathophysiology (15, 24). Thus, recovery of this pathway by carvedilol suggests an endothelial-based mechanism of action of the drug (11). Carvedilol may affect systemic circulation and these changes might be relevant to systemic effect and not specific to the pulmonary vasculature. Notably, biomarkers of cardiovascular function such as NT-proBNP, nitrate, and cGMP were highly correlated with clinical parameters in PH patients at baseline. However, these biomarkers did not predict therapeutic response to carvedilol at any time point.

The short-term response to carvedilol as defined by a drop in RVSP of more than 10 mmHg identified long-term responders with sustained reduction in pulmonary artery pressure at 6-months of therapy. Long-term responders had lower RVSP and PVR on follow up with no change in cardiac output. A favorable response to pulmonary vasodilators and earlier calcium channel blockers has been studied in an attempt to identify PAH patients that would respond long-term to calcium channel blockers (CCB) and carry better prognosis (25–28). During acute vasodilator testing, a minority of patients with idiopathic PAH (~10%) have a more pronounced fall in mean pulmonary arterial pressure and lower absolute values (25). These patients have a sustained long-term hemodynamic benefit to CCB and better long-term survival (25, 29). Here the short-term response to carvedilol predicted 6-months response. Importantly, there were no other differences between responders and non-responders at baseline and markers of disease severity were not different.

The drop in stroke volume and contractility described is similar to what is described in earlier studies in left heart failure (30, 31). In fact, short-term effects of β-blockade in left heart failure are different than long-term effects with an acute depression in systolic function followed by marked improvement with longer use (30). The effects of β-blockers are seen after 3 to 12 months depending on the severity of myocardial dysfunction (31). Here, short-term carvedilol led to a drop in left ventricular stroke volume in PH patients. In non-responders, this was associated with reduced left ventricular end diastolic volume suggesting decreased RV contractility. However, in responders, this was associated with reduced LV ejection fraction. Nonetheless, LV and RV strains did not significantly drop in either group. Findings suggest a dichotomous phenotypic response to carvedilol among a relatively homogeneous group of patients that otherwise could not be discerned by clinical or physiologic measures.

This study has several limitations. One limitation of the study is the lack of invasive hemodynamic measures. We used echocardiography to assess pulmonary pressure and cardiac function and minimized variance by having all echocardiograms and analyses performed by a single expert sonographer. Echocardiography provides a number of variables for evaluating right heart hemodynamics and left heart function (32, 33). Nevertheless, volumetric assessment by 2-D echocardiography may not be as accurate due to geometric assumptions. Hemodynamics would have been the most reliable measure for pulmonary pressures and cardiac output. This secondary *ad-hoc* investigation of the PAHTCH study limits the power of the analyses. The echocardiogram performed at the 1-week visit was a limited study performed to assess safety. However, a single expert sonographer who was blind to the randomization scheme performed all echocardiograms. Another limitation is the sample size. Dividing participants into responders and non-responders led to smaller subgroups in placebo, low-dose and dose-escalating carvedilol groups, decreasing the power for detection of significant changes. We were able to detect differences over time between responders and non-responders who continued on carvedilol when assignment was characterized by any carvedilol therapy (low dose or dose escalation) vs. no therapy (placebo). The difference in weight between responders and non-responders and the possibility of insufficient dosing of carvedilol might have affected the long-term findings. Based on good clinical practice, all patients were started on a low initial dose of 3.125 mg twice daily regardless of weight (34). The carvedilol response may be influenced by weight due to inadequate amount of β-blocker dosing in the obese, but the finding of a signal of response to low dose carvedilol supports a mechanistic link between β-blockade and cardiovascular physiologic response in PH. Notably, there were responders who weighed over 85 kg, and non-responders who weighed < 85 kg, suggesting that the relationship between dose response and weight/BMI is not the only factor in response to carvedilol in this population. Finally, blood was not collected at the 1 week visit and NT-proBNP and more complete arginine metabolites could not be measured. Additional biomarkers mainly anti-inflammatory markers would have added more mechanistic insight into the role of carvedilol in pulmonary hypertension but were not measured.

In summary, low-dose carvedilol decreases RVSP by more than 10 mmHg in more than 50% of patients with pulmonary hypertension within 1 week of therapy. This effect is sustained with carvedilol up to 6 months and is associated with recovery of endothelial L-arginine/nitric oxide signaling pathway.

## Ethics Statement

The Pulmonary Arterial Hypertension Treatment with Carvedilol for Heart Failure (PAHTCH) trial is a double-blinded, randomized, controlled intervention with three arms preceded by an open-label 1-week run-in period (NCT01586156). It was conducted from 2012 to 2016 at the Cleveland Clinic. The Institutional Review Board at Cleveland Clinic approved the study (IRB number # 11-1198). All participants provided informed consent prior to their participation in the study.

## Author Contributions

SF conducted the study, analyzed and interpreted the data, and wrote the manuscript. DS conducted study and collected and analyzed the data. MP performed and read the echocardiography and collected the data. JT read the echocardiography and reviewed the manuscript. HC performed the research experiment and analyzed and interpreted the data. SC collected the data and samples and put in place database. JS recruited the patient. KH conducted the study and reviewed the manuscript. WT analyzed the data and reviewed the manuscript. SE designed the research, analyzed the data, and wrote the manuscript.

### Conflict of Interest Statement

KH has grants and contracts, consult or is on the speaker's bureau of Actelion Pharmaceuticals, Arena Healthcare, Bayer Healthcare, Boehringer Ingelheim, Eiger Pharmaceuticals, Gilead Sciences, Reata Pharmaceuticals, and United Therapeutics. The remaining authors declare that the research was conducted in the absence of any commercial or financial relationships that could be construed as a potential conflict of interest.
