# Supplementary material for: Endothelial Phenotype Evoked by Low Dose Carvedilol in Pulmonary Hypertension
Source: Front Cardiovasc Med. 2018 Dec 12;5:180. doi: 10.3389/fcvm.2018.00180 (PMC6299019; doi:10.3389/fcvm.2018.00180)
Supplement: Supplementary file 1 [file Data_Sheet_1.docx]

**Endothelial Phenotype Evoked by Low Dose Carvedilol in Pulmonary Hypertension**

Supplemental Material

Intended for publication as an online data supplement

**Supplement Figures**

**
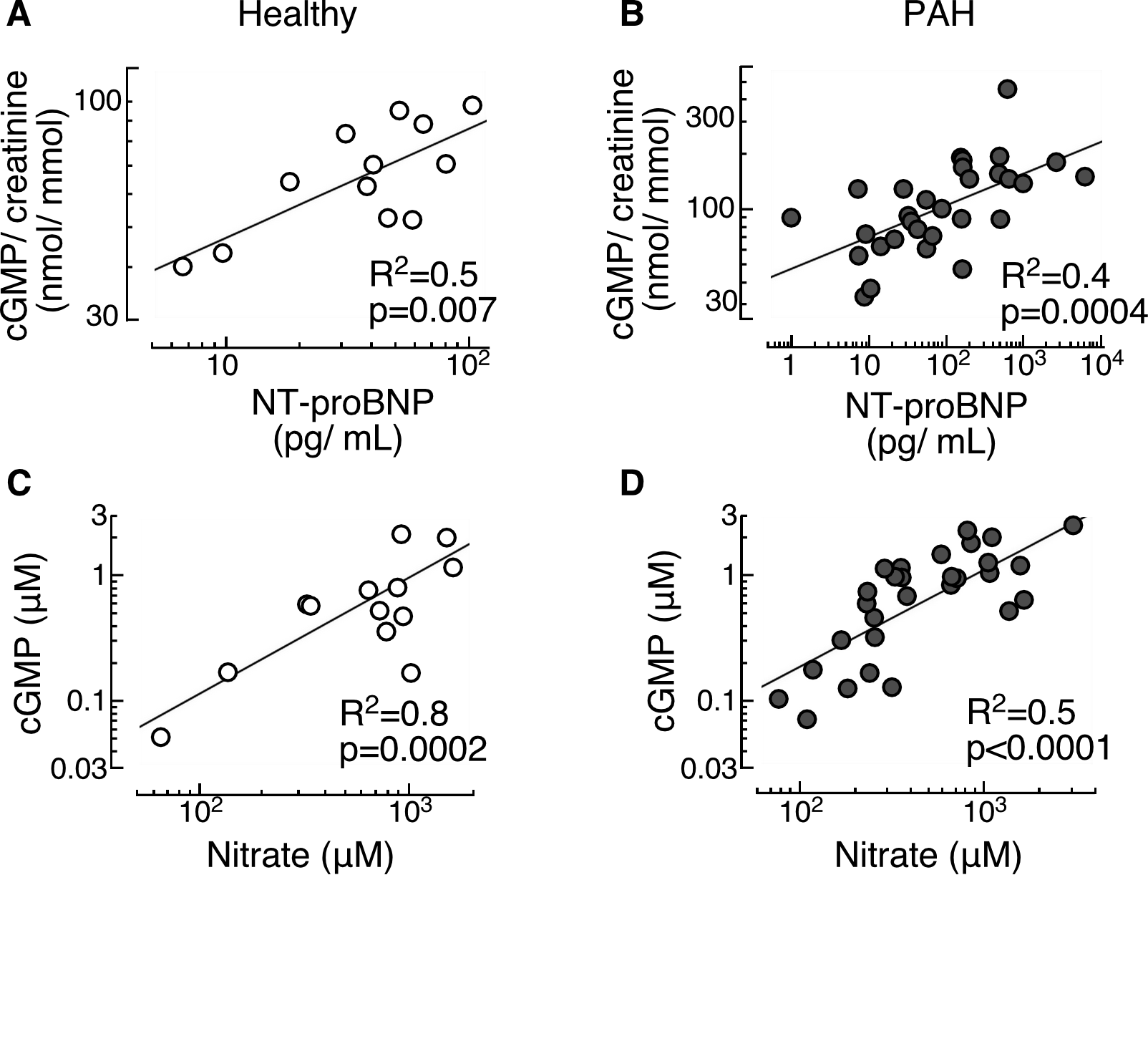
**

**Supplemental Figure 1.** Correlation of urine cyclic guanosine monophosphate (cGMP) with plasma N-terminal pro–B-type natriuretic peptide (NT-proBNP) and urine nitrate. Urine cGMP/ creatinine directly associates with plasma NT-proBNP in healthy (panel A) and PH (panel B) participants. Urine cGMP directly associate with urine nitrate in healthy (panel C) and PH (panel D) participants.

**
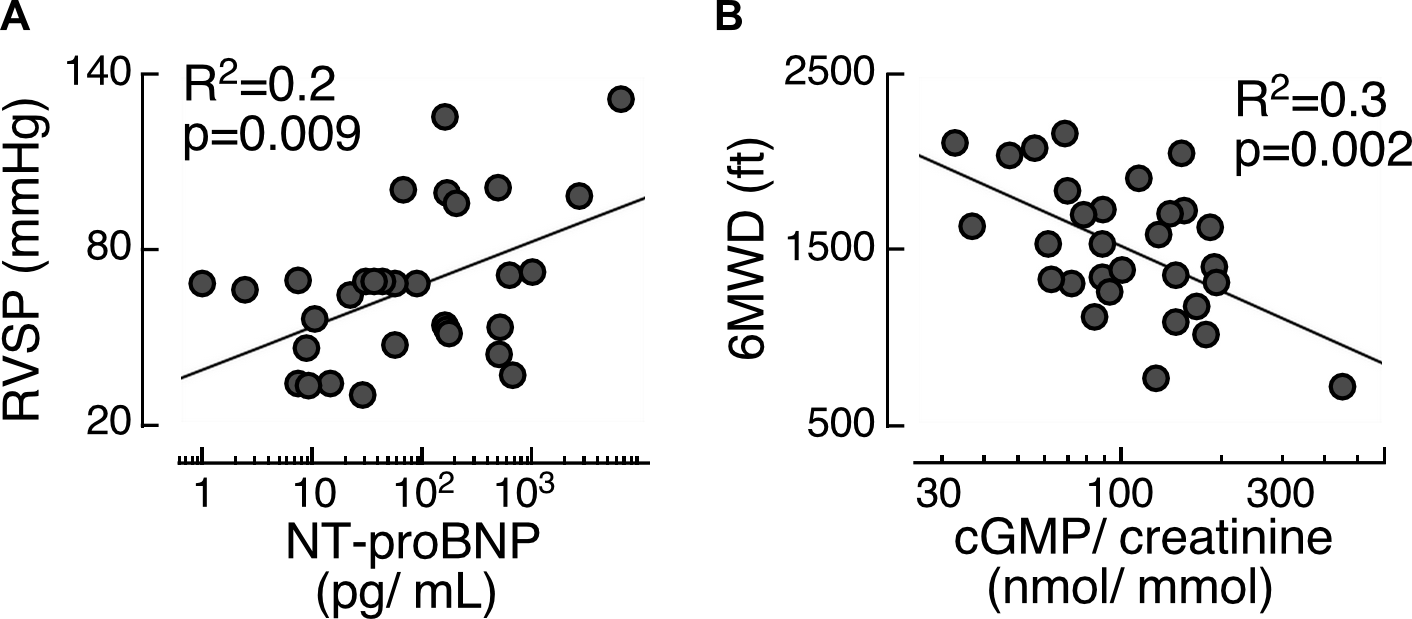
**

**Supplemental Figure 2.** Correlation of right ventricular systolic pressure (RVSP) and 6-minute walk distance (6MWD), with plasma N-terminal pro–B-type natriuretic peptide (NT-proBNP) and urine cyclic guanosine monophosphate cGMP/creatinine in PH. RVSP significantly associates with plasma NT-proBNP (panel A). 6-minute walk distance inversely correlates with urine cGMP/creatinine (panel B).

**
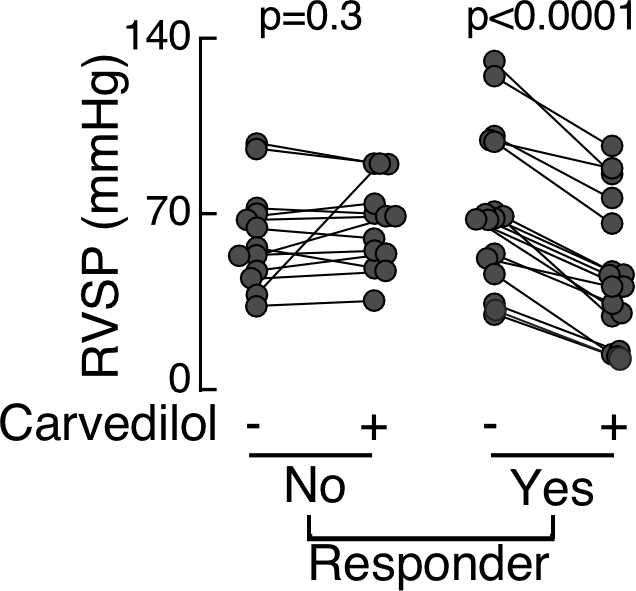
**

**Supplemental Figure 3.** Right ventricular systolic pressure (RVSP) significantly dropped after 1 week of carvedilol 3.125mg twice a day in responders (p < 0.0001). There was no significant change in non-responders (p = 0.3).
